# Biomechanical considerations on tooth-implant supported fixed partial dentures

**DOI:** 10.1177/1758736012462025

**Published:** 2012-10-29

**Authors:** Konstantinos X Michalakis, Pasquale Calvani, Hiroshi Hirayama

**Affiliations:** 1 Division of Removable Prosthodontics, Department of Prosthodontics, School of Dentistry, Aristotle University of Thessaloniki, Thessaloniki, Greece; 2 Division of Graduate and Postgraduate Prosthodontics, Department of Prosthodontics and Operative Dentistry, Tufts University School of Dental Medicine, Boston, MA, USA

**Keywords:** Biomechanics, periodontal ligament, bone, tooth to implant connection

## Abstract

This article discusses the connection of teeth to implants, in order to restore partial edentulism. The main problem arising from this connection is tooth intrusion, which can occur in up to 7.3% of the cases. The justification of this complication is being attempted through the perspective of biomechanics of the involved anatomical structures, that is, the periodontal ligament and the bone, as well as that of the teeth- and implant-supported fixed partial dentures.

Several changes have been made since the introduction of implants for dental prosthetic rehabilitation. The initial concept introduced by Professor Per-Ingvar Brånemark and his associates recommended placement of six implants in the anterior mandibular or maxillary area and construction of a fixed detachable hybrid prosthesis, made of a metal substructure, denture teeth, and heat polymerized acrylic resin material.^1–3^

Since then, researchers and clinicians modified the initial implant treatment options. Implants are now being used for the restoration of maxillary complete edentulism, as well as for the rehabilitation of partial edentulism.^4–7^ Furthermore, connection between teeth and implant fixtures has been advocated as a feasible way to provide prosthetic reconstructions, when anatomical limitations—for example, sinus or mental nerve proximity and lack of sufficient bone quantity—or financial restrictions are present^8,9^ (Figure 1).

**Figure 1.**
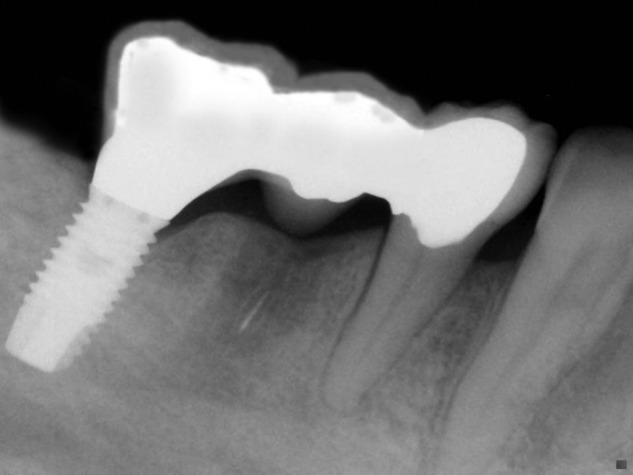
Implant-to-tooth connection with no apparent tooth intrusion.

It should be mentioned that initially separation of the implants from abutment teeth has been recommended, because successfully osseointegrated implant fixtures are practically ankylosed in the bone, while teeth present some mobility due to the presence of the periodontal ligament (PDL). The efficacy of the implant-to-tooth connection has been discussed in the dental literature.^10,11^ Due to the complications (Figure 2) that have been observed, there is an argument among the clinicians regarding the long-term prognoses of these restorations.^12,13^

**Figure 2.**
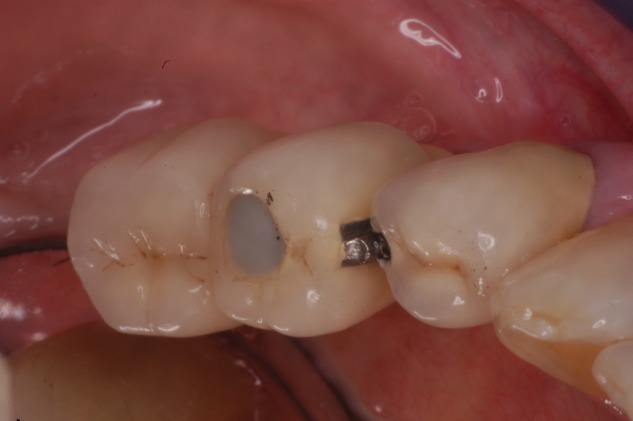
Clinical evidence of evident tooth intrusion in an implant-to-tooth connection. Note the discrepancy at the attachment and at the occlusal surfaces of the restorations supported by the tooth and the implant.

The purpose of this article is to review the existing literature and discuss the biomechanics of the tooth’s supporting tissue (PDL), the biomechanics of implants’ supporting tissue (bone), as well as the biomechanics of the tooth–implant connection, in an attempt to focus on the tooth intrusion phenomenon that is sometimes clinically and radiographically observed.

## Biomechanics of the PDL

The PDL is the connective tissue structure surrounding the root of the tooth and connecting it with the adjacent bone. Its thickness is about 0.2 mm. Histochemical and morphometric analyses of the PDL have demonstrated that it is composed of collagen, oxytalan and elaunin fibers, a ground substance made of glycosaminoglycans, and blood vessels that occupy 4%–47% of the tissue’s volume.^14–19^ Microscopically, the PDL is constructed from fibers with high spatial organization, which can be categorized into six groups: (a) transeptal, (b) alveolar crest, (c) horizontal, (d) oblique, (e) apical, and (f) interradicular (only between the roots of the multirooted teeth).^20,21^ These specialized connective tissue fibers are not unidirectionally distributed. They connect the tooth to the alveolar bone and absorb functional and parafunctional forces originated both from the teeth and other adjacent anatomical structures (i.e. lips, cheeks, and tongue). Another feature that affects the biomechanical response of the PDL is the presence of numerous vessels that exert a hemodynamic pressure.^22–24^ The exact mechanism with which the PDL supports the tooth is not clear.^25^ Three main hypotheses have been made in order to explain the action of the PDL: (a) the *tensional mechanism of tooth support*,^26–28^ which supports the notion that a gradual unfolding of the fibers takes place in order to transmit the loads to the surrounding alveolar bone, (b) the *viscoelastic model*,^29,30^ which considers that tooth displacement is actually controlled by vascular elements and the fibers have a less important function, (c) the *collagenous thixotropic system*, which supports the idea that the PDL possesses thixotropic gel properties.^31,32^

The PDL seems to play an important role in the function of the stomatognathic system by (a) its mechanoreceptors, which transmit to the central nervous system essential information regarding the occlusal forces,^33,34^ (b) furnishing of stimulating tensile loads to the alveolar bone, and (c) the provision of physiologic tooth mobility. This mobility differs among different groups of teeth. Teeth with one root present higher mobility than teeth with multiple roots.^35^ It has also been demonstrated that mobility also varies from day to day or even from hour to hour.^36^ Physiologic teeth mobility can be classified as horizontal and vertical. The upper limit of horizontal tooth mobility has been found to be T_500_ = 0.15 mm for single-rooted teeth and T_500_ = 0.10 mm for multirooted teeth, where T_500_ is the range of tooth movement under an occlusal force of 0.5 kg.^37,38^ Vertical tooth mobility occurs in two stages: (a) the initial or intrasocket stage, in which the tooth moves within the boundaries of the PDL;^39^ this movement occurs with forces of about 100 lb and it ranges between 50 and 100 µm.^40^ (b) The secondary stage, which occurs gradually and entails elastic deformation of the alveolar bone in response to increased horizontal forces.^40^

It has been observed that there is a nonlinear force–displacement relationship for a tooth, regardless of the direction of occlusal loading. Logarithmic relationships between applied forces and teeth displacement have been demonstrated after application of both horizontal and vertical forces.^41,42^ Under the same loading–unloading rates, a hysteresis loop was obtained for axial and horizontal loadings, as well as for rotational movements.^43,44^ However, more recent animal studies have indicated that for occlusal loads between 0.1 and 4.0 N, there is a two-phase parabolic relationship with a discontinuity occurring within the force range of 0.5–0.8 N.^45–49^ These parabolic relationships present the following forms:

$$\begin{array}{c} I=k_{1}\cdot F_{1}^{M},\;\text{for forces between}\;0.1\;\text{and}\;0.5\;\text{N}\\ I=k_{2}\cdot F_{2}^{M},\text{for forces between}\;0.8\;\text{and}\;4\;\text{N} \end{array}$$

where *I* is the intrusion, *F* is the force, and *M*_1_ and *M*_2_ are the slopes of the log displacement versus log force curves. It has also been demonstrated that *M*_1_ is sensitive to the rate of application of occlusal forces, while *M*_2_ is sensitive to periodontal fluid exchanges.

Horizontal forces create greater tooth displacements than vertical loads. It has been shown that a 1-N horizontal force applied for a period of 2 s causes a movement of 150 µm, while the same force applied vertically produces a 15–20 µm displacement. It should be mentioned, however, that tooth movement produced by horizontal loads is also dependent on the duration, rate, and exact point of force application. These horizontal forces can cause buccolingual and mesiodistal movements, as well as extrusion.^50^

A biomechanical analysis of the PDL has been attempted in the past, based on numerical techniques. The formulation of models for the explanation of living material behavior involves the development of relations between stress, strain, and their derivatives with respect to time.^51^ The construction of these constitutive models many times adopted simplifications and assumptions in order to overcome inherent difficulties in obtaining experimental data. In that manner, isotropy and linear elasticity have been incorporated in the mathematical modeling.^52^ However, this simplification may only be used for fairly small stretches, where the real performance of many biological fibers may be sufficiently approached by a linear relationship between force and stretch. In those cases, the stress–strain relationship is expressed by^53^

(1) $$F=\frac{c}{1-\frac{\lambda -1}{\lambda_{c}-1\;}}(\lambda -1)$$

where *c* is an intrinsic property of the fiber, which is independent of the unloaded length of the fiber, *λ* is the stretch parameter, and *λ_c_* is the critical value after which there is a sharp increase of the force in the fiber. For small range extensions, *λ* ≈ 1. Therefore

$$1-\frac{\lambda -1}{\lambda_{c}-1\;}\approx 1$$

and equation (1) becomes

(2) F=c(λ−1)

(2)

The quantity *λ-*1, in equations (1) and (2) is referred to as strain, which can also be expressed as *ϵ*. Thus, equation (2) becomes

(3) F=cε

**Figure 3.**
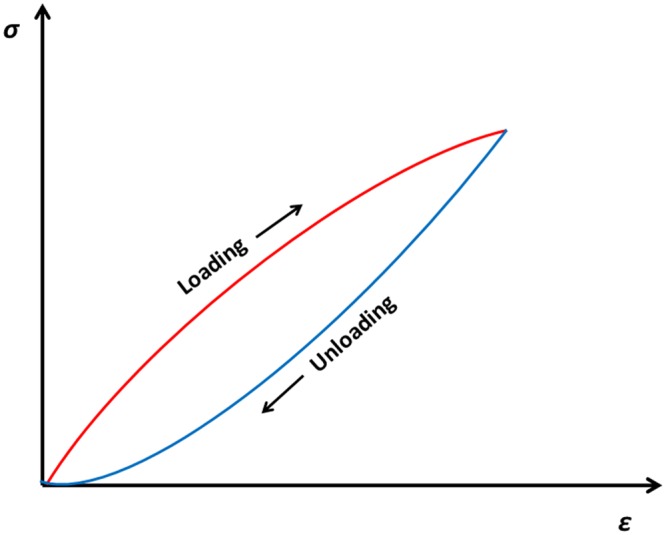
Different paths of loading and unloading of a viscoelastic material.

It should be mentioned, however, that the majority of biologic materials do not demonstrate an elastic behavior, but rather a viscoelastic one. They present the phenomena of both the relaxation and creep. When they are subjected to a cyclic loading, the loading and the unloading processes follow somewhat different paths, which delineate an area representing the amount of energy dissipated as heat during the process (Figure 3). This area is equal to ∮σdε, where *σ* is the stress and *ϵ* is the strain.

The big percentage of water (and other biologic fluids) in the tissues is responsible for their viscoelastic behavior, which in a one-dimensional model is expressed as

(4) $$F=c_{n}\frac{1}{l}\frac{dl}{dt}$$

where $c_{n}$ is the damping coefficient and dl/dt is the time derivative that measures the rate of change of the fiber’s length.

Moreover, the wavy configuration of the PDL’s fibers and the change in their conformation under loading assist the description of the nonlinear stress–strain curve observed, which displays a small stiffness to external loads for moderately big strains and higher and quasi-constant stiffness for bigger strains.^54–56^

There is a question, however, whether one model is sufficient to fully describe the behavior of the PDL, since when a force is exerted on the tooth, some regions of the PDL will always be under compression while others will be under tension.

Experimental data by Bergomi et al.^57^ have demonstrated that the compressive load response of the PDL is viscous, and it may be described by the following pseudoplastic, time-dependent constitutive equation

(5) $$T=-e^{a_{0}+k_{0\lambda }}{\left|\dot{\lambda }\right|}^{n_{0-1}}\left|\dot{\lambda }\right|$$

where *T* is the stress and *λ* is the stretch ratio.

However, this viscous model cannot be assumed for the fibers under tension, since the role of the fluid phase was not found to be equally distributed between the tensile and the compressive parts of the load cycles.^39^ While the results of the compressive phase can be expected to be an interaction amid the porous matrix (fibers, vessels, and ground substance) and the fluid phase (blood and interstitial fluid), the same concept does not seem to apply for the tensile phase. The latter is presumed to be a tissue restructuring, with gradual unfolding, reorientation, and stretching of the collagen fibers.^55,58,59^ Other facts that need to be considered include the movement of the interstitial fluid from compressive to tensile areas, the porosity of the surrounding bone that receives blood and tissue fluids, and the elasticity of the bone that is usually assumed to be negligible due to the big difference with that of the PDL.^60,61^ It has been also postulated that the PDL can be treated as a hyperelastic matrix including numerous collapsible voids. Under compression, these pores can be filled with fluids that in this way can move to nearby areas of the PDL or forced in the adjacent alveolar bone.^62^ This movement of the fluid may also be accompanied by electrokinetic effects, for example, streaming potentials and currents, as the ions circulate through the charged solid medium.^63,64^ The permeability of both the neighboring bone and cementum should be taken into account and can be obtained using Darcy’s relation^65–67^

(6) $$Q=\frac{1}{\mu }\kappa \frac{A\Delta P}{L}$$

where *Q* is the flow rate, *µ* is the dynamic viscosity of the fluid, *κ* is the permeability of the medium, *A* is the cross-sectional area to flow, Δ*P* is the pressure drop, and *L* is the length over which the pressure drop takes place.

In that sense, PDL’s response can be based on Biot’s concept of dynamic poroelasticity and poromechanics, that is, the study of fluid-saturated porous media whose performance on external actions is influenced by the fluid occupying the pores.^62,68,69^ Therefore, the modeling requires a pore-permeated matrix that is filled with a fluid. The solution to this problem involves equations of linear elasticity for the solid medium, Navier–Stokes equations for the fluid, and as mentioned before, Darcy’s equation for the fluid flow. In order to model the solid matrix, Bergomi et al.^62^ have adopted a modification of Ogden’s^70,71^ material model strain energy density

(7) $$\begin{array}{c} W\left(\lambda_{j}\right)=\overset{Ν}{\underset{i=1}{\sum }}\frac{2\mu_{i}}{\alpha_{i}^{2}}\left(\lambda_{1}^{\alpha_{i}}+\lambda_{2}^{\alpha_{i}}+\lambda_{3}^{\alpha_{i}}-3+\frac{1}{\beta_{i}}\left(J^{\alpha_{i}\beta_{i}}-1\right)\right),\\ j=1,2,3 \end{array}$$

where *λ_j_* are the principal stretch ratios; *N, µ_i_*, and *α_i_* are material constants; and *J* is the Jacobian determinant.

It can be understood that this presents a highly complicated problem with blood and lymph vessels, as well as the ground substance forming a continuum arrangement with viscoelastic properties. On the other hand, the collagen fibrils that are embedded in the ground substance alter the symmetry characteristics of the material.^56^ As a result, an anisotropic mechanical response and nonlinear behavior are expected, due to the multidirectional arrangement of the collagen fibrils. The crimped conformation of the fibrils and the anisotropy of the material have led some research groups to adopt a hyperelastic model in order to describe the response of the PDL.^56,72^ In brief, a material can be called hyperelastic or Green elastic when the material’s response is such that the stress power and the stress work may be obtained from a scalar valued potential ϕ=ϕ[E] such that^73^

(8) $$\omega =\dot{\phi }=\frac{\partial \phi }{\partial E}:\dot{E}=\frac{\partial \phi }{\partial E_{ij}}{\dot{E}}_{ij}$$

(9) $$w=\overset{t}{\underset{t_{o}}{\int }}\omega dt={\left[\;\phi \right]}_{t_{0}}^{t}=\phi \left[E\right]-\;\phi \left[E_{0}\right]\equiv \Delta \phi$$

where *ω* is the stress power per unit volume, **E** is the state of strain, ϕ[E] is the elastic energy or strain energy per unit volume, and *w* is the stress work per unit volume, when the material is deformed.

The following equations also hold

(10) $$\omega =\sigma \dot{\varepsilon }$$

(11) $$w=\overset{t}{\underset{t_{o}}{\int }}\omega dt=\overset{t}{\underset{t_{o}}{\int }}\sigma \dot{\varepsilon }dt=\overset{\varepsilon }{\underset{\varepsilon_{o}}{\int }}\sigma d\varepsilon$$

where *σ* is the stress and *ϵ* is the strain.

The deformation of the PDL under functional and parafunctional loads will as a result have the accumulation of some energy, which may be expressed as^56^

(12) W=W(F)

where *F* is the deformation gradient and *W*(*F*) is the strain energy density function. This equation may also be expressed in terms of the principal invariants of the Cauchy–Green tensor as

(13) $$W=W(I_{1},I_{2},I_{3})$$

Taking into account the isotropic contribution of the matrix (*W_m_*), the anisotropic contribution of the fibrils (*W_f_*), and the interaction of these two media (*W_mf_*), the above-described equation may be expressed as

(14) $$W=W_{m}\left(I_{1},I_{2},I_{3}\right)+W_{f}\left(I_{4}\right)+W_{mf}\left(I_{1},I_{2},I_{3},I_{4}\right)$$

It should be mentioned, however, that *W_mf_* is usually omitted. Consequently, the Cauchy stress tensor is given by

(15) $$\sigma =\overset{3}{\underset{i=1}{\sum }}\sigma_{i}\frac{\lambda_{i}}{\lambda_{1}\lambda_{2}\lambda_{3}}\frac{\partial \tilde{W}}{\partial \lambda_{i}}n_{i}⊗n_{i}+\lambda_{4}\frac{\partial \tilde{W}}{\partial \lambda_{4}}n_{4}⊗n_{4}$$

where $\sigma_{i}$ are the principal stresses and *n_i_* the related principal directions of stresses.

The possibility of a progressive failure of the collagen fibrils and the surrounding matrix has also been evaluated in the past and elasto-damage models have been developed to accommodate the behavior of the PDL under large strains.^54,72^ This model considers the failure as irreversible, and it neglects the repairing cellular activity that is overcome by the speed of the mechanical damage process. The deformation of the PDL collagen fibers, until total failure, follows the hierarchical structure: (a) collagen molecular deformation, (b) fibril deformation, and (c) fiber deformation.^74,75^ This implies that the molecular elongation is the first incident in the deformation process, with changes in the *D*-period structure. On a molecular level, a type I collagen molecule is made of three polypeptide chains twisted together to form a helix structure. Studies of Cowan et al.^76^ and Sasaki and Odajima^77^ have demonstrated that the distance *d* between adjacent amino acids can be a measure for the molecular strain *ϵ_d_*

(16) $$\varepsilon_{d}=\frac{\left(d-d_{0}\right)}{d_{0}}$$

where *d*_0_ is the distance between the neighboring amino acids before the force application.

At the fibril level, the distance *D* (~67 nm) between two neighboring molecules can also be a measure for the strain^74,78^

(17) $$\varepsilon_{D}=\frac{\left(D-D_{0}\right)}{D_{0}}$$

where *D*_0_ is the distance between the neighboring amino acids before the force application.

It should be mentioned that the strain observed for a collagen molecule is the strain in the helix pitch. On the other hand, the strain for the *D*-period occurs by a rearrangement of the molecules.^75^ At the higher level of the hierarchy, the collagen fibers are responsible for the tensile response of the PDL. The wavy configuration that the fibers have in the relaxed state makes them unable to resist elongation. Therefore, they cannot offer any mechanical contribution until they are stretched. The number *n* of fibers participating, when a tensile force is exerted and causes a stretch *x*, is^79^

(18) $$dn\left(x\right)=n\frac{1}{\sqrt{2\pi s}}e^{\frac{-{\left(x-\mu \right)}^{2}}{2s^{2}}}dx$$

where *µ* is the mean value of the length of the collagen fibers and *s* is the standard deviation.

When some tensile force is exerted on a tooth, we have to assume that not all PDL fibers will participate immediately. Since all fibers have the same modulus of elasticity and limit strain, the fibers will start failing in the same sequence in which they started participating. According to Haut,^80^ when a fiber is stretched beyond its limit strain (*ϵ_l_*), it will finally rupture, and the number of fibers that participate at a stretch $x/1+\varepsilon_{l}$ is

(19) $$\begin{array}{c} dn\left(\frac{x}{1+\varepsilon_{l}}\right)=\frac{n}{\sqrt{2\pi s}}e^{\frac{-{\left(\frac{x}{1+\varepsilon_{l}}-\mu \right)}^{2}}{2s^{2}}}d\left(\frac{x}{1+\varepsilon_{l}}\right),\\ \text{with}\;x\geq 1+\varepsilon_{l} \end{array}$$

Therefore, the damage function *D_f_* of the PDL fibers is given by^56^

(20) $$D_{f}=\frac{dn\left(\frac{x}{1+\varepsilon_{l}}\right)}{dn\left(x\right)},\;with\;x\geq 1+\varepsilon_{l}$$

In the same manner, a damage of the matrix may also occur due to a combination of creep and cyclic loading. The damage function *D_m_*(*t*) can be found through a continuum damage mechanics approach, where the damage parameter *D_m_* is defined through the use of effective stress $\sigma^{\prime }$^81,82^

(21) $$\sigma^{\prime }=\frac{\sigma }{1-D}$$

where *σ* is the applied stress. It should be mentioned that the net stress is the true stress due to the damage growth, which results in the reduction of the load bearing area (*A_l_*). Consequently, the net stress is based on the net area. For the undamaged matrix *D_m_* = 0 and $\sigma^{\prime }=\sigma$. However, as *D_m_* → 1, *A_l_* → 0 and $\sigma^{\prime }\to \infty$

The development of damage within the matrix can be found using the Kachanov’s^83^ damage growth law

(22) $$\frac{dD_{m}}{d_{t}}=C{\left(\frac{\sigma \left(t\right)}{1-D_{m}}\right)}^{\eta }$$

where *C* and *η* are the material constants.

The damage function of the matrix is

(23) $$Dm\left(t\right)=1-{\left\{1-C(\eta +1)\overset{t}{\underset{0}{\int }}[\sigma {\left(t\right)}^{\eta }]dt\right\}}^{\frac{1}{\eta +1}}$$

The damage of the matrix and fibers results in a degenerative condition of the PDL, which may be a key factor in the intrusion phenomenon sometimes observed when teeth are connected with implants.

## Biomechanics of the bone

The bone is composed of mineral (65%), organic matrix (35%), cells, and water. The organic matrix is basically made of collagen creating a meshwork with spaces filled with the bone mineral that is in the form of crystals. These crystals come in different shapes, such as plates, rods, and needles, and are basically impure hydroxyapatite.^84^

The residual bone of the maxilla and the mandible is the bone of the alveolar process that remains after the extractions of the teeth. It consists of cortical and trabecular (cancellous) bone. These two different types of bone are different in many aspects, including development, architecture, function, blood supply, rate of turnover time, as well as the degree of age-dependent changes.^84–86^ The cortical bone is very dense, with a porosity percentage of 3%–5%. It has microscopic channels and it can be further classified as primary or secondary. The primary cortical bone consists of highly organized lamellar layers, which have a thickness of 3–7 µm containing fine fibers that run approximately in the same direction. It should be mentioned, however, that in neighboring lamellae, these collagen fibers may have different orientations.^87,88^ On the other hand, the secondary cortical bone consists of layers that are disrupted by tunnels of osteons. The trabecular (cancellous) bone is formed by intersecting calcified tissue (trabeculae) and presents a porous structure, with porosity up to 90%.^89^ It can be understood that bone presents an anatomical structure that is hierarchically organized with an irregular arrangement and orientation of its components. As a result, bone presents heterogeneity and anisotropy.^90^ It has been demonstrated that the mechanical properties of bone vary at different levels. However, it is not known whether these differences are due to the influence of the microstructure or due to the application of different research methods.

The modulus of elasticity of the bone has been shown to be related to its density^91–93^

$$E=3790{έ}^{0.06}\rho^{3}$$

where *E* is the modulus of elasticity (in MPa), is the strain rate (in s^−1^), and *ρ* is the density of the bone (in g cm^−3^). This equation applies for samples including both cortical and trabecular bones. Research on trabecular bone specimens revealed that the modulus of elasticity and the strength are proportional to the square of apparent density of the tissue and are therefore proportional to one another.^94^

Although the exact properties of the cancellous bone have not been clearly identified, there is strong evidence that Young’s modulus and the response of the trabecular bone to compressive and tensile loadings are quite different from those of the cortical bone. A correlation between the density and the compressive strength in trabecular bone has been established^93^

$$\sigma^{-}=68{έ}^{0.06}\rho^{2}$$

where *σ^−^* represents the compressive strength, is the strain rate (in s^−1^), and *ρ* is the density of the bone (g cm^−3^).

Therefore, the suggestion of Wolff^95^ that compact bone is simply more dense cancellous bone tissue is not an accurate statement when only the mechanical properties of these two tissues are considered. In fact, it seems that the proximity as well as the orientation of the trabeculae regulates the mechanical properties of cancellous bone.^96^ Other factors influencing the properties of the bones include the age, sex, metabolic and hormonal functions, and location (i.e. femur, jaw, etc).

The cortical–trabecular bone proportion can vary greatly between individuals. A jaw bone quality classification has been proposed by Lekholm and Zarb:^97^ type 1: almost the whole bone consists of homogenous cortical bone, type 2: a small core of dense trabecular bone is surrounded by thick cortical stratum, type 3: a big core of dense trabecular bone is surrounded by a thin layer of cortical bone, and type 4: a big part of trabecular bone of low density is surrounded by thin cortical stratum.

Cortical bone is considered an anisotropic material, due to the presence of rigid hydroxyapatite-like mineral particles that are embedded in a collagen fiber matrix. According to Fratzl et al.,^98^ the anisotropic shapes of the mineral particles are responsible for the anisotropy presented in the mechanical properties of the cortical bone. In general, cortical bone presents a viscoelastic behavior.^99^ Initially, under a load application to the bone, an elastic reaction is observed. Subsequently, a strain that develops and increases over time is seen. The rate of the strain depends on the magnitude of the force. It has been demonstrated that the strain decreases towards zero, after the removal of the load. This indicates that the bone demonstrates a passive creep behavior. Tennyson et al.^100^ found that the stress–strain relationship of post-mortem bovine femoral cortical bone obeys to the Kelvin–Voigt model equation of the form:

(24) $$\sigma =E\varepsilon +\eta \dot{\varepsilon }$$

where *E* is a long-term elastic constant and *η* is the viscosity.

The viscoelastic behavior of the cortical bone was shown to be related to its hydration state. Therefore, a reduction in the water content has been proven to result to a significant decrease of the amount of viscoelasticity of the human femoral cortical bone.^101^ Another study by Adharapurapu et al.^102^ has demonstrated that the modulus of elasticity of dry cortical bone specimens is lower than that of rehydrated specimens.

**Figure 4.**
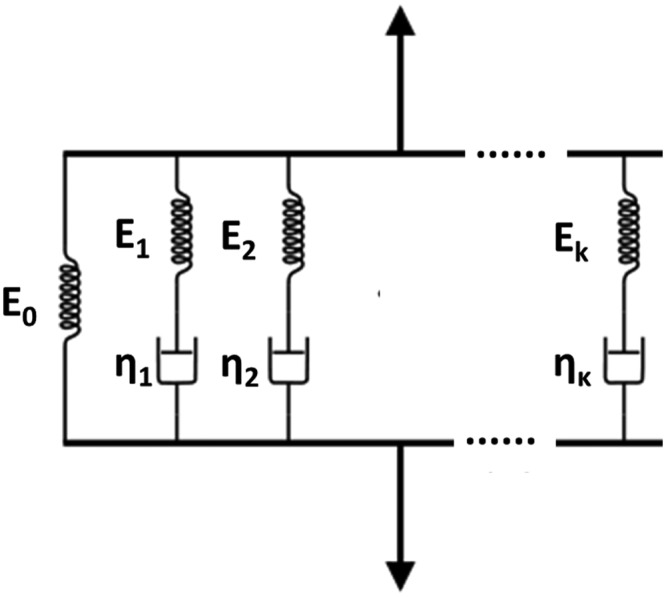
Illustration of a general form of the Maxwell–Wiechert model.

The Maxwell–Wiechert model (Figure 4) can also be used in order to accommodate a distribution of relaxation times due to different molecular segment lengths, which as a result have a faster relaxation time for shorter segments than for the longer ones.^103^ In that model, the total stress equals the stress of the isolated spring plus the sum of the stresses of all the Maxwell spring–dashpot arms

(25) $$\sigma =\sigma_{e}+\underset{j}{\sum }\sigma_{j}$$

It should be mentioned, however, that previous research has shown that a linear viscoelastic theory with a single relaxation mechanism does not have an application to bone.^101,104^ Therefore, constitutive models with both viscoelastic and viscoplastic components have been proposed in order to explain the behavior of the cortical bone. In that model, the viscoelastic component can be described by the following equation, which contains two linear viscoelastic mechanisms, namely 1 and 2^105^

(26) $${\dot{\varepsilon }}^{VE}=\frac{\sigma (t)}{\left(E_{0}t+\eta_{1}\left(1-e^{-\frac{E_{1}t}{\eta_{1}}}\right)+\eta_{2}\left(1-e^{-\frac{E_{2}t}{\eta_{2}}}\right)\right)}$$

where *VE* is the viscoelastic strain and 0 is used for the long-term equilibrium behavior.

The viscoplastic behavior of the cortical bone may be obtained from the dependence of yield stress on strain rate, from the work of McElhaney,^106^ as well as that of Crowninshield and Pope,^107^ and is given by the following equation

(27) $${\dot{\varepsilon }}^{VP}=\frac{\sigma }{\left|\sigma \right|}{\left(\frac{\left|\sigma \right|}{S_{0}}\right)}^{k}$$

where *k* is calculated from the inverse of the slope of the trend of the stress–strain curve, while *S*_0_ is the natural logarithm given by the stress axis intercept.

The final viscoelastic–viscoplastic model may be obtained from equations (26) and (27), where the total strain rate is given by

(28) $$\dot{\varepsilon }={\dot{\varepsilon }}^{VE}+{\dot{\varepsilon }}^{VP}$$

The porous structure of the cancellous bone as a result has a different mechanical behavior than that of the cortical bone. The existence of trabecular rods–plates and the pores that are filled with a viscous fluid form a matter with a complex mechanical response during loading.^108–110^ The big difference between the bulk modulus of the solid and the fluid phases, as well as the minute local deformations, as a result has a biphasic mechanical response to external loads, which may be expressed by a poroelastic model.^61,110^ Trabecular bone is an anisotropic material and a reformation of the equations of anisotropic poroelasticity has been presented by Thompson and Willis.^111^ However, for simplicity reasons, the equations presented by Cowin^61^ were for an isotropy case, and the isotropic stress–strain relationship is expressed as

(29) $$2G\varepsilon_{ij}=\sigma_{ij}-\left(\frac{\nu }{1+\nu }\right)\sigma_{kk}\delta_{ij}+\alpha \left(\frac{1-2\nu }{1+\nu }\right)p\delta_{ij}$$

while the stress–strain relations are

(30) $$\sigma_{ij}+\alpha p\delta_{ij}=2G\varepsilon_{ij}+\left(\frac{2Gv}{1-2\nu }\right)\varepsilon_{kk}\delta_{ij}$$

where *σ_ij_* represents the components of the total stress tensor, *ϵ_ij_* represents the components of the strain tensor in the solid phase, *p* is the pore pressure, *G* and *v* are the shear modulus and Poisson’s ratio of the trabecular bone under the drained condition, respectively, and *α* represents the ratio of the fluid ratio changes due to the volume discrepancies of the bone when it is loaded under drained conditions.

It has been demonstrated that bone under the application of a cyclic load can develop microcracks that can ultimately cause fatigue failure. It should be mentioned, however, that these phenomena have been observed in vitro.^112,113^ There is data indicating that under normal conditions, these microcracks do not necessarily lead to failure. On the contrary, a remodeling process can be initiated in order to overcome the potential destructive effects of the cycling loading. It should be mentioned that strain seems to be the key factor between loading forces and bone remodeling. It has been found that the mass of bone is controlled by an equilibrium between resorption and bone formation, which are controlled by the endocrine—and the mechanically produced—drive, respectively. There is a requirement of a few cycles of dynamic loading for an osteogenic response.^114^

Besides the mechanical damage accumulation, a biological one may also take place. According to Nash,^115^ this is expressed as

(31) $$D_{T}=D_{S}-H+D_{D}+D_{A}$$

where *D_T_* is the total damage at any time, *D_S_* represents the damage due to a general stress, *H* is the damage that is repaired by the healing process, *D_D_* is the damage due to disease, and finally *D_A_* is the damage caused by the aging process. This model applies to cyclic loading cases, and *D_S_* is calculated from the following equation^116^

(32) $$D_{S}=D_{F}=\overset{m}{\underset{i=1}{\sum }}{\left(\frac{n}{N}\right)}_{i}$$

where *N_i_* represents the number of loading cycles that is required to cause failure at the *i*th stress level, *n_i_* is the number of cycles executed at the *i*th stress level, and *m* represents the number of significant stress levels.

Carter and Caler^116^ have presented a model of stress-related bone damage in order to provide a comprehensive model of the damage–repair process in living bone. This model assumes that within the bone, there is a progressive accumulation of cracks that is related to a damage function *D_c_*, which takes values between 0 and 1. When *D_c_* has a value of 0, there is no creep damage, and when *D_c_* = 1, a creep fracture occurs. It can be understood that damage accumulation is a time-dependent process, and the cumulative creep damage due to cycle loading is

(33) $$D_{c}\left(t\right)=\overset{t}{\underset{0}{\int }}\left(\frac{1}{k\sigma {\left(t\right)}^{-c}}\right)dt$$

where *k* and *c* are the constants.

It has been demonstrated that during fatigue loading, an internal damage takes place that results in a progressive loss of bone stiffness and strength that eventually leads to failure.^117^ Another study by the same group has concluded that the type of load, that is, tensile or compressive, has a different effect on the type of damage observed. Tensile fatigue load creates a widespread failure at the cement lines, as well as at the interlamellar cement bands. On the other hand, a compressive fatigue load produces several oblique microcracks that are influenced by stress concentrations created by lacunae and vascular canals.^118^ The properties of the cortical and the trabecular bones determine their behavior to the application of mechanical loads. Application of a compressive cyclic load as a result has a greater bone deposition in cortical bone than when a tensile cyclic load is applied.^113^ This phenomenon is due to different generated electric potentials in bone. Bone compression as a result has generation of a negative electric potential, while a tensile load produces positive potentials.^119^

An insight into the complicated processes of bone adaptation has been attempted by McNamara et al.,^120^ who have claimed that bone remodeling is initiated by accumulative damage. An assumption has been made that even at the remodeling equilibrium, some damage exists, and the repair rate is related to the damage rate

(34) $$\frac{dX}{dt}=C\cdot \omega_{eff}$$

where *dX* is the repair rate, *dt* is the damage rate, *C* represents the remodeling coefficient, and *ω_eff_* represents the effective damage of the bone.

A model that includes the cellular activity within the bone has also been described:^121,122^

(35) $$\dot{d}=\lambda_{b}a_{b}n_{b}-\lambda_{c}a_{c}n_{c}$$

where *λ* is the surface area fraction available, *a* represents cellular activity, *n* represents cellular number, while *b* and *c* represent osteoblastic and osteoclastic activity, respectively. This theory was recently tested by a research group and gave encouraging results.^123^

## Biomechanics of tooth-implant-supported fixed partial dentures

It can be seen that bone exhibits a completely different biomechanical behavior than the PDL. Connection of teeth, which present some mobility, with implants, which are practically ankylosed in the bone, presents some risks that should be taken into account before making such a decision.

A careful treatment planning should be made by assessing the following biomechanical factors:

- Mobility of the teeth to be connected with implants.
- Number of teeth and implants to be connected.
- Occlusal forces including
  (a) magnitude
  (b) duration
  (c) distribution
  (d) direction

- Rigidity of the prosthesis.
- Type of connection (rigid or nonrigid).
- Type of the bone.

### Mobility of teeth to be connected with implants

As mentioned before, periodontally healthy teeth have a physiologic mobility due to the PDL. Different groups of teeth present different degrees of mobility. Posterior teeth usually present less mobility than anterior teeth. Canines display less mobility than incisors. On the other hand, implants present no mobility. Clinical research has indicated that the Periotest^®^ values^124,125^ of successfully osseointegrated fixtures in the mandible range from −4 to −2. Mandibular canines present values between −1 and +4.^126^ The higher damping values found at implants have been attributed to the absence of the PDL. Another reason accounting for these values is that titanium’s elastic modulus is 110–117 GPa^127^ as compared to dentin’s, which is 12–14.7 GPa.^128^ Young’s modulus of adjacent anatomical structures, such as cortical and trabecular bones, as well as that of the PDL, is important, but it will be discussed later on.

There is controversial information in the dental literature regarding the periodontal status of the teeth to be connected to implants. Kindberg et al.^13^ have reported that treatments with periodontally sound teeth and implants splinted together in one-piece prostheses with rigid connections show excellent long-term results. Nevertheless, Cordaro et al.^129^ have shown that when teeth are connected to implants with a rigid connection, the intrusion phenomenon is more likely to happen to teeth with an intact periodontal support than to teeth with a reduced periodontal support.

The position of the teeth—to be connected to implants—in the arch does not seem to be associated with the intrusion phenomenon.^8^ However, the proximity of the tooth to the implant is probably important and it will be discussed in the next section.

### Number of teeth and implants to be connected

Information on how the number of teeth connected with implants influences tooth intrusion is scarce. A retrospective multicenter study, which investigated the complications arising from the connection of teeth with implants, has reported that the intrusion of the supporting teeth was most common in the prosthesis design with one implant connected to one tooth.^12^ This finding was based on an up to 3-year follow-up of 220 abutment teeth connected with 185 implants in 111 patients. The authors of the aforementioned study^12^ found that the intrusion of natural teeth retainers was 5%, while 72.72% of this was attributed to a one-to-one implant-to-tooth connection. However, another clinical study^8^ that evaluated 339 implants connected to 313 teeth in 123 patients for up to 15 years reported an incidence of tooth intrusion in 7.31% of the cases. Prostheses supported by one tooth and one implant accounted for 44.44% of the total intrusion cases. An interesting point of this study, which is not mentioned by the authors, regards cases in which multiple natural teeth are connected to implants. The data support the notion that in the majority of the cases that developed an intrusion, the intrusion was found in natural teeth abutments that were adjacent to the implants.

### Occlusal forces

#### Magnitude

The magnitude of chewing and maximum biting forces varies considerably among the individuals.^130–132^ It should be mentioned, however, that in the majority of the patients, the chewing forces are smaller than the maximum biting forces. Normally, when the individual has a fixed complete denture, chewing forces range between 55 and 165 N, while maximum biting forces can usually be between 264 and 336 N. The latter can very rarely reach values of 4340 N.^133^ Biting forces are influenced by many factors such as type of restorations, premature contacts, skeletal and anatomical factors and parafunctional habits. It has been proved by clinical studies that the forces are noticeably different whether the fixed partial dentures are supported by natural teeth or implants. The presence of bilateral terminal abutments and bilateral or unilateral cantilevers is also important for the biting force magnitude. Existence of natural teeth or a complete denture on the opposing arch also plays a role.^134^ The presence of premature contacts, even if these are as small as 100 µm, can increase the biting forces substantially.^135,136^

Skeletal factors may influence the magnitude of chewing and maximum biting forces. It has been demonstrated that subjects with low Frankfort mandibular plane angle (FMA) can produce almost twice as much occlusal forces, in the molar region, when compared to individuals with high FMA.^137,138^ The anatomy of the masseter muscle, especially its cross-sectional area, can also influence the biting force exerted in the molar area.^139^ Parafunctional habits such as clenching and bruxism may affect both the magnitude and the duration of the forces.

#### Duration

Occlusal forces are exerted on the teeth and/or on the restorations, both while swallowing and chewing. During the waking state, occlusal contacts from swallowing occur about every 2 min.^140^ During sleep, the occlusal contacts due to swallowing are irregular and much less frequent.^141^ It has been estimated that from swallowing alone, opposing teeth touch about 1500 times each day.^142^

Prolonged duration of occlusal contacts is observed in subjects with parafunctional habits such as bruxism and clenching. A study has shown that subjects with parafunctional activities occlude their teeth seven times longer than those with no such habits (38.7 min vs 5.4 min).^143^

#### Distribution

Distribution of forces on occluding units has an impact on both muscular contraction and the mechanical effect on each tooth.^144^ However, the outcome of uneven force distribution is different for anterior and posterior teeth. For anterior teeth, the greatest proprioceptive inhibition results if only one tooth is in contact with its antagonist.^145^ The problem arises from the fact that in that scenario, all the muscular contractions and the resultant forces will be placed on that tooth, with detrimental effects. Posterior teeth are quite different from anterior ones in the sense that an occlusal contact on a tooth stimulates the muscles to their greatest level of contractibility. Therefore, existence of a single posterior occlusal contact does not trigger the proprioception to stop muscle contraction. As a result, the posterior tooth will take all the potential force that the elevator muscles can generate. The aim of the clinicians should be an even distribution of biting forces in maximum intercuspation to as many posterior teeth as possible.^146^ Nevertheless, definite occlusal concepts have not been developed regarding implant prostheses.^132^ Even worse, when the treatment involves both natural teeth and implants, furnishing of a specific occlusal scheme is empirical, since the literature on this subject is scarce.

Whether or not equal distribution of occlusal forces between natural teeth and implants is mandatory is not known yet. The dissimilar physical characteristics of the PDL and the bone as a result have a different behavior of teeth and implants when subjected to forces. Under a 20-N force, teeth will usually intrude 50 µm,^28^ while implants will intrude about 2 µm under the application of the same force.^147^ As a result, if occlusal equilibration is performed in such a way as to have an equal distribution of forces under light contacts, the implants will be subjected to a force overload when heavier occlusal contacts will be exerted, since the natural teeth will intrude into their sockets while implants will not. Consequently, in a tooth-implant fixed partial denture, as the tooth will intrude, the prosthesis will act as a cantilever to the implant. On the other hand, if the equilibration is performed under heavy occlusal contacts, the forces will be evenly distributed between teeth and implants.^148^ The number of splinted teeth and implants, mobility of periodontally involved abutment teeth, crown to implant ratio, quality of the bone where the implant was placed, location of the fixed tooth-to-implant prosthesis in relation to the elevator muscles and parafunctional activities are important factors that have to be evaluated when the occlusal equilibration is performed.

#### Direction

Vertically directed occlusal loads can be beneficiary to the teeth, since they do not induce excessive mobility that lateral loads can create.^149,150^ One of the goals of occlusal adjustment is the redirection of forces along the long axes of the teeth. According to the experimental data and biomechanical calculations, osseointegrated implants should ideally be loaded axially.^151^ Off-axial forces due to erroneous implant placement, wrong prosthesis design, or mediotrusive (nonworking) contacts will create bending moments given by the following equation^152^

(36) M=F⋅L

where *M* is the bending moment (in N m), *F* is the force (in N), and *L* represents the lever arm (in m). These bending moments will be transferred from the prosthesis to the implant and will ultimately end in the bone.

It should be mentioned that in tooth-to-implant fixed prosthesis, even axial directed forces can create bending moments to the implant, if the natural tooth has excessive mobility or if it intrudes. These forces will be applied to the prosthesis, the implant prosthetic components (i.e. fastening screw and abutment), the implant itself, and the bone that is in close contact to the fixture.

### Rigidity of the prosthesis

Fixed partial or complete dentures should be made with certain dimensions to ensure minimal flexure, which is essential for the longevity of the restoration. A thickness of 0.3 mm for base metal alloys and 0.5 mm for high noble alloys, as well as 3 × 3 mm^2^ sized connectors are essential to obtain a quite rigid prosthesis.^153^ This concept has been used successfully in traditional prosthodontics for many years. Rigidity of the metal substructure guarantees that there will be no fracture of the overlying esthetic material (acrylic or porcelain) and no cement wash out at the abutments. However, whether the connection between implants and teeth should be rigid or not is not resolved yet. This clinical approach is quite new and is still under investigation, because of frequent complications. These include fracture of implant components, damage, and/or intrusion of the abutment teeth.^154^

Several theories have been developed in order to explain the tooth intrusion phenomenon:

1. *Disuse atrophy*. The fibers of the PDL of the tooth may undergo a disuse atrophy due to the hypofunction of the tooth, since the implant undertakes the majority of the occlusal forces.^155–157^
2. *Differential energy dissipation*. There is an osteoclastic activity in the PDL due to very high stress transmitted to the tooth.^158,159^ The result of this osteoclastic activity is the intrusion of the abutment tooth.
3. *Impaired rebound memory*. This theory suggests that due to the constant pressure, the PDL loses its elastic memory and remodels in a new position. This position is more apical than the original one. The PDL’s remodeling continues until the tooth is completely out of occlusion and stabilizes in that new position.^160–162^
4. *Rachet effect*. The abutment tooth moves apically due to the occlusal overload and stays in that new position, maybe because of the binding in the socket or in the semiprecision attachments that are very often used.^161,162^
5. *Debris impaction*. Food particles can entrap under telescopic copings or in the connecting attachments and prevent the tooth to return to the original position.
6. *Fixed prosthesis flexure*. All beams flex under a stress. Similarly, a fixed prosthesis can flex and force the abutment tooth out of its original position into the socket.
7. *Mandibular flexure*. This theory applies only to mandibular tooth-to-implant prostheses.^163–168^ Mandibular flexure is a phenomenon that occurs when the mouth opens, due to the contraction of the muscles of mastication and the flexibility of the mandibular bone. This flexure is clinically observed as a posterior narrowing. Therefore, teeth and implants change their relative positions and an internal stress is generated. This repeated stress can result in the abutment tooth movement.

Fabrication of a rigid connection between implants and teeth was questioned in the past because of the differences in their supporting tissues. Skalak’s^169^ theory involved lateral stiffness, torsion and bending of the prosthesis, and bending or rotation of the fastening screws and other prosthetic components. According to this hypothesis, distribution of the forces largely depends on the stiffness of the interacting units. The stiffnesses involved are lateral, torsional, and bending.

When a lateral force *F* is applied to a unit (regardless whether it is an implant or a natural tooth), it causes a deflection *δ*_1_. The elastic constant of this unit in lateral stiffness is $K=F/\delta_{1}$. Stiffness of an implant is bigger than that of a natural tooth, mainly because of the different physical characteristics between the bone and the PDL. Use of a compliant material was suggested in order to level the behavioral differences of the two dissimilar units and obtain an equal load distribution and a deflection ${\overset{\prime }{\delta }}_{1}$ that is less steep than *δ*_1_.

Torsional and bending stiffnesses are associated with torque and bending moments in the fixed prosthesis and the way these are transferred to the supporting units (teeth or implants). This requires a very complex analysis due to the fact that the torsional stiffnesses of the implants and teeth as well as the geometric curvature of the prosthesis should also be taken into account. However, Skalak^169^ believed that incorporation of a compliant material into the prosthesis could provide beneficiary long-term results as the stiffnesses, the implants, and the teeth would be in equilibrium.

Use of polyoxymethylene as a compliant material, to absorb a big part of the forces applied to the implant and resemble in this way the action of the PDL, has been suggested.^170–174^ However, clinical data indicated that this material did not serve successfully its purpose and required frequent replacement.^175,176^

### Type of connection (rigid or nonrigid)

Connection of teeth with implants may take place either with an attachment system or with a telescopic crown. This type of connection is dictated by the need of implant prostheses retrievability for reservicing, replacement, or salvaging of the restorations and the implants.

Attachment systems connecting two parts of a fixed prosthesis can be either rigid or nonrigid. In rigid connectors, there is usually a fastening screw that fixes the patrix and the matrix parts rigidly. In nonrigid attachments, there is a key and a keyway part that slide one into the other, but there is no screw to fix these two parts. Likewise if, instead of an attachment system, a telescopic crown is used, then this can be either fixed rigidly to the suprastructure with a screw or with a definitive cement.

Clinical studies have shown that a rigid type of connection should be preferred, since with the use of a nonrigid connection, an intrusion may occur in 3%–4% of the cases.^8,12^ Nevertheless, a finite element analysis and photoelastic study have demonstrated that with a rigid type of connection, more stress is concentrated around the implant.^177^ Likewise, a radiographical study has indicated that more bone loss is observed in the area around implants when the tooth–implant connection is rigid.^9^

### Type of the bone

An area that has not been investigated at all is related to the type of the bone and its contributory effects to the tooth–implant connection. As stated by Lekholm and Zarb,^97^ there are four different types of bone, with variations in the cortical and trabecular bone contents. The elastic moduli of cortical and trabecular bones are quite different. The first one has a Young’s modulus of 15,000 MPa, while the cancellous bone has a modulus of elasticity of 1500 MPa.^178–181^ The PDL has an elastic modulus of 1.18–2 MPa.^52,182^ It should be mentioned, however, that the bone values mentioned do not take into account differences that may occur due to variations of primary and secondary cortical bones, as well as differences due to the density and the organization of the trabeculae in cancellous bone. Research has demonstrated that the mechanical properties and the elastic modulus of the bone are influenced by these factors.^94,96^ Consequently, differences in the bone type and its architecture—at the microscopic level—may influence the outcome of tooth–implant connection. Research is required in order to confirm or reject this hypothesis.

## Conclusion

Clinicians should be aware of the limitations and disadvantages of the teeth connected to implant prostheses, so that they can plan prosthetic treatments accordingly. The risks related to teeth connected to implants prostheses result from differences in the biomechanics of the involved anatomical structures (i.e. the PDL and the bone) and of the biomechanics of teeth-implants-supported fixed prostheses.
